# Effects of Alternate Insulin Pump Settings in Patients With Type 1 Diabetes During Ramadan: A Randomized Pilot Study

**DOI:** 10.1177/19322968211059217

**Published:** 2021-11-22

**Authors:** Ghufran AlGhatam, Derek O’Keeffe, Husain Taha

**Affiliations:** 1 Department of Medicine, National University of Ireland Galway, Galway, Ireland; 2 Salmaniya Medical Complex, Manama, Bahrain; 3 University Hospital Galway, Galway, Ireland

**Keywords:** continuous glucose monitoring, insulin pump, Ramadan fasting, time in range, type 1 diabetes

## Abstract

**Background:**

Various studies have evaluated the safety and efficacy of using insulin pumps during Ramadan; some of them demonstrated favorable outcomes in reducing hypoglycemia and hyperglycemia. However, there is no consensus on the recommendations for basal insulin adjustments and the utilization of technical features of insulin pumps to improve glycemic control.

**Objectives:**

We aimed to investigate the effects of different insulin pump settings on time in range in patients with type 1 diabetes during Ramadan.

**Methods:**

In this randomized pilot study, 30 patients classified to have low to moderate risk for fasting were assigned to either a control group to receive basal insulin adjustments only or an intervention group to use the temporary basal rate and extended bolus features in addition to the basal insulin modifications. The percentage of time spent at different glucose ranges was measured by continuous glucose monitoring.

**Results:**

The percentage of time spent within target (70-180 mg/dL) increased significantly in the intervention group from 63.0 ± 10.7 to 76 ± 16.2% (mean difference, 27% points; *P* < .001). The percentage of time spent in hyperglycemia level 1 (>180 mg/dL) and level 2 (>250 mg/dL) met the criterion of significance, indicating that the intervention group spent less time in hyperglycemia. However, there was no significant difference in the percentage of time spent in hypoglycemia ranges.

**Conclusions:**

Incorporating technological approaches of pump therapy with clinical practice guidelines could improve glycemic control during Ramadan.

## Introduction

Ramadan fasting (RF) is one of the five pillars of Islam. Muslims are refrained from eating, drinking, smoking, and sexual intercourse, and abstained from consuming any oral medications, starting from sunrise until sunset for 29 to 30 days each year. Around 1.8 billion Muslims worldwide devote themselves to fasting during Ramadan annually^1^ because it is a compulsory deed of worship for all healthy Muslims after puberty. Nevertheless, individuals with chronic conditions, including some people with diabetes (PWD), are religiously and medically exempted from fasting. Despite this, the estimated number of fasting Muslims with diabetes is at least 50 million globally,^2,3^ and many insist on fasting sometimes against the medical recommendations.

Ramadan fasting entails distinctive changes in food and fluids consumption^4^ that could potentially induce metabolic alterations in glucose metabolism and insulin sensitivity.^5-7^ There are also inter-individual variabilities in the glycemic parameters, which could be attributed to the cultural differences, dietary patterns, and the fasting duration across different geographical regions.^6,8^ In PWD, the process of glucose hemostasis is complex due to the pharmacokinetics and pharmacodynamics of different medications, including insulin. Patients who fast, especially those with type 1 diabetes mellitus (T1DM), are predisposed to excessive glycogenolysis, gluconeogenesis, and increased ketogenesis,^9-11^ resulting in increased risk for hypoglycemia, hyperglycemia, diabetic ketoacidosis, and dehydration.^10-12^

Still, only a few studies investigated glucose excursions during Ramadan using continuous glucose monitoring (CGM).^13-15^ These studies suggest that RF causes higher rates of hyperglycemia than hypoglycemia, as CGM profiles revealed typical patterns of a rapid spike after iftar (sunset meal) that last overnight, followed by a second rise after the suhoor (pre-dawn meal), with prolonged glucose decline over fasting hours.

Individuals with T1DM who are fasting throughout Ramadan constitute a unique population. Continuous subcutaneous insulin infusion (CSII) is an established therapy option offered for this category. This form of therapy has several advantages over multiple dose injection therapy for fasting individuals because of the capability to adjust insulin doses according to the individual’s physiological requirements during fasting hours. In addition, sensor augmented pumps (SAPs) are an advanced form of CSII therapy, which controls insulin delivery by a glucose sensor with a relevant algorithm. Sensor augmented pumps’ superiority to traditional CSII has been demonstrated in randomized controlled clinical trials; therefore, these devices provide innovative protection against the risks associated with fasting. A cluster of studies revealed the safety of using CSII therapy to reduce hypoglycemia^2^,^16-18^ and improve glycemic variability^17,18^ during RF. Although these data suggest the favorable effects of insulin pumps on glucose control, achieving glycemic control and time-in-range [TIR] goals during Ramadan remain challenging for many patients.^19^

There is a dearth in the research field examining the optimal clinical uses of insulin pump technology in diabetes management during RF, and very few studies have evaluated TIR in SAPs treated patients during Ramadan. In the present study, we propose that the potential variabilities in glucose patterns can be further managed by expanding the use of different pump technology features. The temporary basal rates (TBR) and extended bolus (EB) options were used to investigate their effect on TIR among T1DM patients during RF.

## Methods

### Selection and Description of Participants

**Figure 1.**
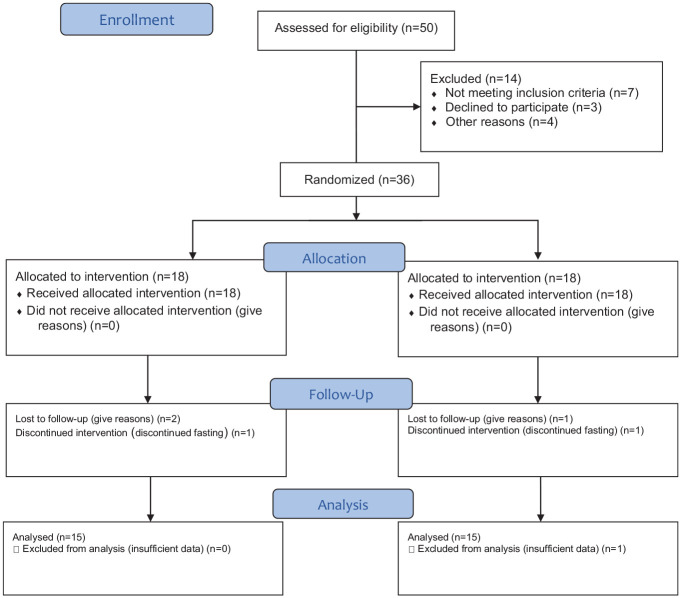
Consort flow diagram.

Thirty individuals participated in this study, who were selected from the outpatient clinics at Salmaniya Medical Complex, which is the largest public secondary and tertiary care hospital in Bahrain. We recruited men and women above 18 years old with T1DM who use SAP therapy (MiniMed 640G) with Guardian Link and Enlite 2 sensor (Medtronic, USA). The patients intended to fast during the month of Ramadan between April 13, 2021, and May 12, 2021. The inclusion was limited to participants with sufficient technical knowledge to communicate with the research team online due to Covid-19 restrictions (Figure 1). All candidates received pre-fasting assessment using the International Diabetes Federation–Diabetes and Ramadan (IDF-DAR) risk stratification calculator, which is a scoring system to assess fasting eligibility for PWD by considering various factors affecting fasting^20^; only participants with low to moderate risk were included. The exclusion criteria included pregnancy, diabetes complications, history of severe hypoglycemia, or diabetic ketoacidosis in the last six months. Volunteers with high fasting risk scores were also excluded. An informed consent form was obtained from all participants, and the hospital’s research ethics committee approved the study (SHCRSC Ref. No. 24250221).

### Experimental Protocol

This study used the IDF-DAR 2021 practice guidelines for diabetes management in CSII users. Both groups received general education about fasting and healthy eating habits during Ramadan. According to each participant’s individual needs and pre-Ramadan glucose control, basal insulin was reduced by 20% to 40% in the last four hours of fast and increased by 10% to 30% in the first three hours of iftar. The bolus insulin ratio remained unchanged as before Ramadan. The smartguard feature, which suspends insulin when sensor glucose approaches a pre-defined low limit (65 mg/dL), was activated for both groups to prevent hypoglycemia during fasting. The data from the devices were downloaded and reviewed. Throughout the trial, participants were encouraged to report any serious adverse events like pump malfunctioning, severe hypoglycemia (defined as hypoglycemia that necessitates other person’s assistance due to altered consciousness), and hyperglycemia with ketones.

### The TBR Feature

Patients in the intervention group received additional training on TBR feature to adjust basal insulin by ±10%-30% for two to three hours to optimize glucose control with recurrent hypoglycemia or suspend before low before breaking the fast or with persistent hyperglycemia two hours post-iftar.

### The EB Feature

Further education was delivered to the intervention group about the optimal utilization of EB delivery in the form of the dual-wave bolus, which delivers insulin instantly followed by an extended delivery over several hours, to match insulin delivery with the high in fat and protein content of traditional meals. Participants were instructed to administer the EB 10 minutes before the meal as a 50%:50% or 60%:40% bolus: square-wave over two hours, according to the glucose reading pre-meal and meal composition.

### Outcomes

Time in range is a parameter that evaluates glucose control by the percentage of time a person with diabetes spends within the target range of 70 to 180 mg/dL. The primary outcome of this study is the time spent within the target range (70-180 mg/dL), which was measured before and after RF and compared between the two study groups. The secondary outcomes were the average glucose and the percentage of time spent in the hypoglycemic range defined as level 1 (<70 mg/dL) and level 2 (<54 mg/dL), and percentage of time spent in hyperglycemic range in level 1 (>180 mg/dL) and level 2 (>250 mg/dL).

### Statistics

The SPSS v27 software (SPSS Inc, Chicago, IL) was used to perform the statistical analyses. The descriptive statistics are presented as mean ± standard deviation (SD) and percentages, depending on data distribution. All variables were tested for normal distribution by the Shapiro-Wilk test, Levene’s test, and Box’s test. The mixed-design analysis if variance (ANOVA) test and the independent *t*-test were used to compare the difference in the percentage of time spent at different glucose ranges between the two study groups. A *P* value < .05 was considered statistically significant, and all tests were two-tailed.

## Results

A total of 36 patients were recruited, and 30 completed the study between April 10, 2021, and May 15, 2021. They were randomly assigned to either the control group (18 patients) or the experimental group (18 patients); the statistical analysis includes data for the participants who completed the study (15 participants in each group; Figure 1). In all, 42% of the participants were men, the patients’ mean age was 22.4 ± 3.9 years, and baseline glycated hemoglobin level ranged from 5.5% to 9.3%, with a mean of (7.5% ± 1.1). The baseline characteristics of the study group are listed in Table 1.

**Table 1.** Characteristics of the Patients.

Characteristic	Overall	Control (n = 12)	Experiment (n= 12)	*P* value*
Age	22.4 ± 3.9	22.6 ± 4.2	22.2 ± 3.8	NA*
Sex: n (%)				
Female	NA*	7 (58%)	7 (58%)	NA*
Male	NA*	5 (42%)	5 (42%)	NA*
HbA1c %	58.5 ± 12.1 mmol/mol	63.9 ± 9.8 mmol/mol	53 ± 12 mmol/mol	NA*
	7.5% ± 1.1	8% ± 0.9	7% ± 1.1	
FD	26 ± 3.9	25 (83%)	28 (93%)	.07
PPH events	8 ± 5.4	12 ± 4.9	5 ± 3.2	<.001

Abbreviations: NA, not applicable; HbA1c, glycated hemoglobin at baseline; FD, number of fasting days during Ramadan; PPH events, number of post-prandial hyperglycemia events.

* *P* value is for analysis with independent *t*-test.

**Figure 2.**
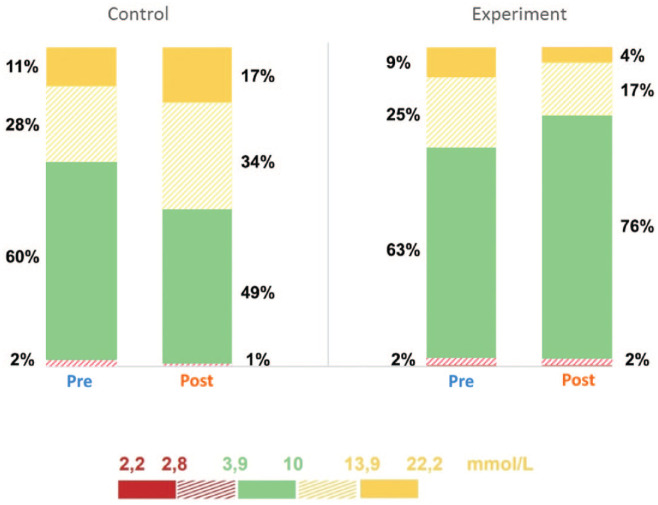
Comparison between different glucose ranges pre- and post-intervention.

The CGM recordings were processed in accordance with international consensus recommendations on the use of CGM.^21^ This study included data for 14 consecutive days before fasting and 30 days during the study period in Ramadan with a minimum of 70% of CGM data capture. The mean glucose improved significantly in the intervention, whereas it declined in the control group at the end of Ramadan. The percentage of time spent within the target range (70-180 mg/dL) increased significantly in the intervention group from 63.0% ± 10.7% at baseline to 76% ± 16.2% at the end of Ramadan (*P* < .001). Conversely, the percentage of time spent in the level of the hyperglycemic range 1 (>180 mg/dL) and level 2 (>250 mg/dL) reduced significantly (*P* < .001). Moreover, no significant difference was observed between the groups in the percentage of time spent in the hypoglycemic ranges (Figure 2). There was a slight reduction in the total daily dose of insulin at the end of Ramadan comparing with before fasting total dose; however, the difference was not found to be significant when compared between groups (Table 2).

**Table 2.** Significance Tests of Different Glucose Ranges.

Variable	Time	Means ± SD			Independent *t*-test		Mixed-design ANOVA		
		Study group			Difference (experimental – control) (95% CI)	*P* value*	Tests of within-subjects effects	Tests of between-subjects effects	
		Control	Experiment	Total			Time	Time × study group	Study group
Hypo level 2	Pre	0.2 ± 0.6	0.4 ± 0.7	0.3 ± 0.6	0.3 (−0.9 to 0.2)	.207	0.460	0.804	0.147
	Post	0 ± 0	0.3 ± 0.9	0.2 ± 0.6					
Hypo level 1	Pre	1.8 ± 1.3	2.4 ± 1.7	2.1 ± 1.5	1.3 (−2.2 to −0.85)	.074	0.040	0.260	0.068
	Post	0.8 ± 0.8	2.1 ± 1.2	1.4 ± 1.2					
TIR	Pre	59.7 ± 7.8	63.0 ± 10.7	61.4 ± 9.3	27 (40 to 15)	<.001	0.541	<0.001	0.004
	Post	48.7 ± 12.7	76.2 ± 16.2	62.4 ± 20					
Hyper level 1	Pre	27.6 ± 4.6	25.0 ± 6	26.3 ± 5.4	−16 (9 to 23.8)	<.001	0.431	<0.001	0.002
	Post	33.5 ± 6.7	17.1 ± 10.4	25.3 ± 12					
Hyper level 2	Pre	10.7 ± 6.5	9.2 ± 6.9	9.9 ± 6.6	−13 (6 to19.6)	<.001	0.462	<0.001	0.019
	Post	17.1 ± 9.2	4.3 ± 6.4	10.7 ± 10.2					
Mean glucose	Pre	186.5 ± 25.2	183.5 ± 22.9	185 ± 23.6	(27.5 to 74.1)	.000	0.650	0.000	0.010
	Post	214.5 ± 36.3	160.7 ± 27.6	187.6 ± 41.8					
Average insulin	Pre	59.5 ± 15.9	64.6 ± 21.4	62 ± 18.6	(−4.3 to 7.5)	.576	0.024	0.576	0.569
	Post	56.9 ± 13.8	60.3 ± 21.1	58.6 ± 17.6					

Abbreviations: CI, confidence interval; Hypo level 2, hypoglycemia range (<54 mg/dL); Hypo level 1, hypoglycemia range (<70 mg/dL); TIR, time at target range (70-180 mg/dL); Hyper level 1, hyperglycemia range (>180 mg/dL); Hyper level 2, hyperglycemia range (>250 mg/dL).

* *P* value < .05.

### Adverse Events

The participants did not report any adverse events during the study period; however, one patient from the control group reported pump malfunctioning, which was replaced on the same day. Neither group reported any events of severe hypoglycemia nor diabetic ketoacidosis during Ramadan. The mean of post-iftar hyperglycemia events was statistically significant (*P* value < .001) among the control and the experimental group 12 ± 4.9 versus 5 ± 3.2, respectively, in which about 21% of all participants (of which one event occurred in the experimental group) reported the need to take exogenous insulin dose to correct level 2 hyperglycemia (>250 mg/dL). The number of hyperglycemia events is associated with the number of fasting days as all participants were instructed to break their fast when blood glucose values are below 70 mg/dL or above 300 mg/dL, even though participants in the experimental group fasted more days 28 ± 2.8 versus 25 ± 4.5, the difference was not statically significant.

## Discussion

The current study met its primary and secondary endpoints, demonstrating that people with T1DM treated with insulin pump therapy could safely improve the percentage of time spent within the target range without an increase in hypoglycemia or significant hyperglycemia, if they use the TBR and EB features to adjust insulin according to their requirements during the month of Ramadan.

Ramadan fasting creates changes in lifestyle, causing biochemical alterations that affect the overall glucose management, even in the presence of best practice guidelines. Our study proved that fasting during Ramadan is associated with radical changes in glucose profiles and TIR. We showed that the percentage of time spent within 70 to 180 mg/dL was notably prominent in the experimental group who received our intensified treatment protocol. Glucose at target range increased 27% points with the advanced technology use compared over the use of standard practice guidelines. There were observed increases in glucose overall, which occurs mostly after iftar meal, yet significant reductions were also seen in hyperglycemic ranges in the intervention group when compared with controls.

Previous CGM studies on insulin-treated patients have reported that RF is associated with an increased percentage of time spent in hyperglycemia and a reduced percentage of time spent in the target range.^19,22^ Nonetheless, several studies reported fewer hypoglycemia episodes and improved glycemic variabilities among CSII-treated patients,^2,17^ which could be attributed to the benefits of reducing basal insulin infusion rates or suspending it to avoid hypoglycemia episodes during fasting.^16^

Time in range has been recently approved as an outcome measure for glycemic control in clinical trials,^23^ which enhances the effectiveness of CGM metrics by establishing treatment goals for the patients. Therefore, it could help in understanding the physiological changes associated with RF, as well as facilitating patients’ education on how to overcome glucose control barriers linked to Ramadan rituals. According to the International Consensus on CGM, each 5% increase in TIR is associated with clinically significant benefits for individuals with T1DM.^21^ Therefore, the 27% points increase in TIR shown in our study has clinical relevance in that it would correspond to improved glucose management during Ramadan. There was also a significant difference in the mean glucose and the hyperglycemic ranges among the groups, which was mostly but not solely repeated after the iftar meal, despite the basal insulin increments during the fast-breaking period. The risk of hypoglycemia, which is of more clinical concern, was prominently low, with the basal insulin reduction in the last four to five hours of fasting, in the two groups.

The glucose variability is of paramount importance in glycemic management as emerging evidence suggests the potential association with several acute and chronic complications.^19,24^ Therefore, improving post-prandial hyperglycemia should be considered a strategy for preventing and managing diabetes complications.^24,25^ In this study, the experimental group experienced less average glucose and lower hyperglycemia episodes than the control group in both hyperglycemic levels 1 and 2. We speculate that the superior clinical outcomes in our study were probably driven by the benefits of using TBR and EB features for glucose management and reduction of post-iftar hyperglycemic events during Ramadan. This is supported by the fact that the number of post-iftar hyperglycemic events that occurred between 6 and 9 pm was considerably greater among participants in the control group, in which patients did not use these features and spent more time in the hyperglycemic range.

Other studies that investigated the impact of EB delivery on glycemic control have reported that the dual-wave bolus feature is particularly helpful to prevent prolonged post-prandial hyperglycemia resulting from the consumption of meals high in fat and protein.^26,27^ Klupa et al^30^ have shown that frequent users of dual-wave bolus achieved improvements in HbA1c levels by 0.45% (*P* = .0009) in two years of clinical observation. Similarly, Chase et al^31^ verified that the dual-wave bolus was effective in achieving lower glucose levels four hours post-prandial with high carbohydrate, fat, and calories consumption. Consistently, our results advocate that dual-wave bolus can be an effective method for optimizing post-prandial glucose during Ramadan. Although there are no clear guidelines for administering dual-wave bolus for different meals, experts recommend extended insulin delivery for meals rich in complex carbohydrates, fat, and protein,^28^ which is the case in Ramadan’s meals composition.

The TIR improvements seen in our protocol that employs different predictors affecting glucose are similar to that seen in a previous study, involving 150 participants aged 5 to 20 years treated with CSII therapy, in whom HbA1c decreased with the use of TBRs (*P* = .01).^26^ This feature is very useful to manage unplanned high or low blood glucose levels resulting from exercise or other events.^29^ Such findings suggest that patients may likely benefit from TBRs to achieve a higher TIR during Ramadan, while basal insulin adjustments are still required from the health care provider before starting the fast.

In addition, patients’ education has been identified as an important component to enhance glucose control during Ramadan.^32,33^ Al-Ozairi et al^34^ showed that patients with T1DM, whether treated with injections or CSII, who underwent diabetes education involving Dose Adjustment for Normal Eating (DAFNE) and basal insulin reduction in a controlled fashion, were able to improve TIR during Ramadan. Correspondingly, consolidating practice guidelines with structured education on optimal clinical uses of insulin pump technology during Ramadan allows properly trained patients to monitor their glucose levels and adjust basal insulin infusion and/or insulin delivery according to carbohydrate consumption and meals composition to avoid hypoglycemia or hyperglycemia in Ramadan.

To our knowledge, this was the first study that examined the effects of employing different insulin pump settings to manage glucose during Ramadan. Importantly, the presented study included patients treated with SAPs who represent a particular category of T1DM patients. Therefore, the current findings provide insight into how to advise and manage this category of patients to avoid glycemic excursions associated with RF. Some of the strengths that contributed to the favorable outcomes in our study include the careful selection of participants, the patient retention rate, and the structured education session delivered before Ramadan that highlighted food choices usually consumed throughout Ramadan, which empowered participants to adhere to the assigned treatment protocols. Nevertheless, our study has certain limitations, including the small patient number and the wide range of lifestyle factors that might affect the results, which are beyond what we have covered in this study.

The SAPs provide a wide range of features for diabetes management, which could lead to better glycemic control when considering the dietary alterations encountered during Ramadan. Despite the small number of participants, the safety outcomes in this cohort were novel and promising. Extended bolus delivery and TBRs may indicate a decreased percentage of time spent in the hyperglycemic ranges and an increased percentage of time spent in the target range during the month of Ramadan. Yet, randomized controlled trials are needed to validate these findings.

## Conclusions

The present study created promising clinical data on glucose management during Ramadan in patients with T1DM treated with insulin pumps. It endorses the importance of following practice guidelines to optimize glycemic control during Ramadan. Nevertheless, our data add to the existing body of evidence and provide support for reviewing the current therapy guidelines. Our findings also point that current practice guidelines can incorporate technological approaches like TBRs and EB delivery to support glycemic control during the Ramadan fast. This study further emphasizes the influential role that health care providers can play in educating patients on how to fully benefit from all pump features during the month of Ramadan.

## References

[1] Lipka M . Muslims, and Islam: key findings in the U.S. and around the world. Pew Research Center. https://www.pewresearch.org/fact-tank/2017/08/09/muslims-and-islam-key-findings-in-the-u-s-and-around-the-world/. Published 2017. Accessed June 8, 2021.

[2] Al-Arouj M Assaad-Khalil S Buse J , et al. Recommendations for management of diabetes during Ramadan: update 2010. Diabetes Care. 2010;33(8):1895-1902. doi:10.2337/dc10-0896. 20668157 PMC2909082

[3] Saeedi P Petersohn I Salpea P , et al. Global and regional diabetes prevalence estimates for 2019 and projections for 2030 and 2045: results from the International Diabetes Federation Diabetes Atlas, 9(th) edition. Diabetes Res Clin Pract. 2019;157:107843. doi:10.1016/j.diabres.2019.107843. 31518657

[4] Alamoudi R Alsubaiee M Alqarni A , et al. Attitudes and habits of patients with type 1 diabetes during fasting Ramadan. J Clin Transl Endocrinol. 2018;14:1-4. doi:10.1016/j.jcte.2018.09.001. 30294552 PMC6169503

[5] Afandi B Kaplan W Al Hassani N Hadi S Mohamed A. Correlation between pre-Ramadan glycemic control and subsequent glucose fluctuation during fasting in adolescents with Type 1 diabetes. J Endocrinol Invest. 2017;40(7):741-744. doi:10.1007/s40618-017-0633-y. 28239763

[6] Kaplan W Afandi B. Blood glucose fluctuation during Ramadan fasting in adolescents with Type 1 diabetes: findings of continuous glucose monitoring. Diabetes Care. 2015;38(10):e162-e163. doi:10.2337/dc15-1108. 26294662

[7] Lessan N Saadane I Alkaf B , et al. The effects of Ramadan fasting on activity and energy expenditure. Am J Clin Nutr. 2018;107(1):54-61. doi:10.1093/ajcn/nqx016. 29381798

[8] Vasan SK Karol R Mahendri NV Arulappan N Jacob JJ Thomas N. A prospective assessment of dietary patterns in Muslim subjects with type 2 diabetes who undertake fasting during Ramadan. Indian J Endocrinol Metab. 2012;16(4):552-557. doi:10.4103/2230-8210.98009. 22837915 PMC3401755

[9] Karamat MA Syed A Hanif W. Review of diabetes management and guidelines during Ramadan. J R Soc Med. 2010;103(4):139-147. doi:10.1258/jrsm.2010.090254. 20382905 PMC2853405

[10] Hassanein M Al-Arouj M Hamdy O , et al. Diabetes and Ramadan: practical guidelines. Diabetes Res Clin Pract. 2017;126:303-316. doi:10.1016/j.diabres.2017.03.003. 28347497

[11] Meo SA Hassan A. Physiological changes during fasting in Ramadan. J Pak Med Assoc. 2015;65(5, suppl 1):S6-S14. 26013791

[12] Salti I Bénard E Detournay B , et al. A population-based study of diabetes and its characteristics during the fasting month of Ramadan in 13 countries: results of the epidemiology of diabetes and Ramadan 1422/2001 (EPIDIAR) study. Diabetes Care. 2004;27(10):2306-2311. doi:10.2337/diacare.27.10.2306. 15451892

[13] Lessan N Hannoun Z Hasan H Barakat MT. Glucose excursions and glycaemic control during Ramadan fasting in diabetic patients: insights from continuous glucose monitoring (CGM). Diabetes Metab. 2015;41(1):28-36. doi:10.1016/j.diabet.2014.11.004. 25497966

[14] Alabbood MH Ho KW Simons MR. The effect of Ramadan fasting on glycaemic control in insulin dependent diabetic patients: a literature review. Diabetes Metab Syndr. 2017;11(1):83-87. doi:10.1016/j.dsx.2016.06.028. 27402028

[15] Alfadhli EM. Higher rate of hyperglycemia than hypoglycemia during Ramadan fasting in patients with uncontrolled type 1 diabetes: insight from continuous glucose monitoring system. Saudi Pharm J. 2018;26(7):965-969. doi:10.1016/j.jsps.2018.05.006. 30416354 PMC6218385

[16] Bin-Abbas BS. Insulin pump therapy during Ramadan fasting in type 1 diabetic adolescents. Ann Saudi Med. 2008;28(4):305-306. doi:10.5144/0256-4947.2008.305. 18596395 PMC6074355

[17] Alamoudi R Alsubaiee M Alqarni A , et al. Comparison of insulin pump therapy and multiple daily injections insulin regimen in patients with type 1 diabetes during Ramadan fasting. Diabetes Technol Ther. 2017;19(6):349-354. doi:10.1089/dia.2016.0418. 28296467

[18] Elbarbary NS. Effectiveness of the low-glucose suspend feature of insulin pump during fasting during Ramadan in type 1 diabetes mellitus. Diabetes Metab Res Rev. 2016;32(6):623-633. doi:10.1002/dmrr.2781. 26789012

[19] Saadane I Ali T El-Laboudi A Lessan N. Ramadan fasting in insulin-treated patients is associated with potentially unfavourable changes in glucose metrics: a flash glucose monitoring (FGM) study. Diabetes Res Clin Pract. 2021;172:108592. doi:10.1016/j.diabres.2020.108592. 33310126

[20] Hassanein M Afandi B Al-Arouj M Shaikh S . Risk stratification of people with diabetes before Ramadan. In: Diabetes and Ramadan: Practical Guidelines 2021. Brussels: International Diabetes Federation and DAR International Alliance; 2021: 91-97.

[21] Danne T Nimri R Battelino T , et al. International consensus on use of continuous glucose monitoring. Diabetes Care. 2017;40(12):1631-1640. doi:10.2337/dc17-1600. 29162583 PMC6467165

[22] Saadane I Ashraf T Ali T Lessan N. Diabetes and Ramadan: utility of flash-glucose monitoring derived markers of glycaemic control and comparison with glycosylated haemoglobin. Diabetes Res Clin Pract. 2019;153:150-156. doi:10.1016/j.diabres.2019.05.020. 31150718

[23] Gabbay MAL Rodacki M Calliari LE , et al. Time in range: a new parameter to evaluate blood glucose control in patients with diabetes. Diabetol Metab Syndr. 2020;12(1):22. doi:10.1186/s13098-020-00529-z. 32190124 PMC7076978

[24] Ceriello A. Glucose variability and diabetic complications: is it time to treat? Diabetes Care. 2020;43(6):1169-1171. doi:10.2337/dci20-0012. 32434893

[25] Ceriello A. Postprandial hyperglycemia and diabetes complications. Is it time to treat? Diabetes. 2005;54(1):1-7. doi:10.2337/diabetes.54.1.1. 15616004

[26] Wilkinson J McFann K Chase HP. Factors affecting improved glycaemic control in youth using insulin pumps. Diabet Med. 2010;27(10):1174-1177. doi:10.1111/j.1464-5491.2010.03068.x. 20854386

[27] Burdick J Chase HP Slover RH , et al. Missed insulin meal boluses and elevated hemoglobin A1c levels in children receiving insulin pump therapy. Pediatrics. 2004;113(3, pt 1):e221-e224. doi:10.1542/peds.113.3.e221. 14993580

[28] Lee S Cao M Sajid S Hayes M Choi L Rother C. The dual-wave bolus feature in continuous subcutaneous insulin infusion pumps controls prolonged post-prandial hyperglycemia better than standard bolus in Type 1 diabetes. Diabetes Nutr Metab. 2004;17:211-216. 15575341

[29] Pańkowska E Szypowska A Lipka M Szpotańska M Błazik M Groele L. Application of novel dual wave meal bolus and its impact on glycated hemoglobin A1c level in children with type 1 diabetes. Pediatr Diabetes. 2009;10(5):298-303. doi:10.1111/j.1399-5448.2008.00471.x. 19175902

[30] Klupa T Skupien J Cyganek K Katra B Sieradzki J Malecki MT. The dual-wave bolus feature in type 1 diabetes adult users of insulin pumps. Acta Diabetol. 2011;48(1):11-14. doi:10.1007/s00592-009-0173-9. 20063022

[31] Chase HP Saib SZ MacKenzie T Hansen MM Garg SK. Post-prandial glucose excursions following four methods of bolus insulin administration in subjects with type 1 diabetes. Diabet Med. 2002;19(4):317-321. doi:10.1046/j.1464-5491.2002.00685.x. 11943004

[32] Al-Agha AE Kafi SE Zain Aldeen AM Khadwardi RH. Flash glucose monitoring system may benefit children and adolescents with type 1 diabetes during fasting at Ramadan. Saudi Med J. 2017;38(4):366-371. doi:10.15537/smj.2017.4.18750. 28397942 PMC5447188

[33] Mohamed K Al-Abdulrazzaq D Fayed A , et al. Fasting during the holy month of Ramadan among older children and adolescents with type 1 diabetes in Kuwait. J Pediatr Endocrinol Metab. 2019;32(8):843-849. doi:10.1515/jpem-2019. 31318694

[34] Al-Ozairi E El Samad A Al Kandari J Aldibbiat AM. Intermittent fasting could be safely achieved in people with type 1 diabetes undergoing structured education and advanced glucose monitoring. Front Endocrinol. 2019;10:849. doi:10.3389/fendo.2019.00849. PMC6906269 31866948

